# Hierarchical nanoporous metals as a path toward the ultimate three-dimensional functionality

**DOI:** 10.1080/14686996.2017.1377047

**Published:** 2017-10-05

**Authors:** Takeshi Fujita

**Affiliations:** ^a^ WPI Advanced Institute for Materials Research, Tohoku University, Sendai, Japan

**Keywords:** Nanoporous metal, dealloying, electrode, catalysis, molecular detection, porous graphene, 3D printing, 60 New topics / Others, 102 Porous / Nanoporous / Nanostructured materials, 205 Catalyst / Photocatalyst / Photosynthesis, 206 Energy conversion / transport / storage / recovery, 207 Fuel cells / Batteries / Super capacitors, 503 TEM, STEM, SEM

## Abstract

Nanoporous metals prepared via dealloying or selective leaching of solid solution alloys and compounds represent an emerging class of materials. They possess a three-dimensional (3D) structure of randomly interpenetrating ligaments/nanopores with sizes between 5 nm and several tens of micrometers, which can be tuned by varying their preparation conditions (such as dealloying time and temperature) or additional thermal coarsening. As compared to other nanostructured materials, nanoporous metals have many advantages, including their bicontinuous structure, tunable pore sizes, bulk form, good electrical conductivity, and high structural stability. Therefore, nanoporous metals represent ideal 3D materials with versatile functionality, which can be utilized in various fields. In this review, we describe the recent applications of nanoporous metals in molecular detection, catalysis, 3D graphene synthesis, hierarchical pore formation, and additive manufacturing (3D printing) together with our own achievements in these areas. Finally, we discuss possible ways of realizing the ultimate 3D functionality beyond the scope of nanoporous metals.

## Graphical abstract

**Figure:**
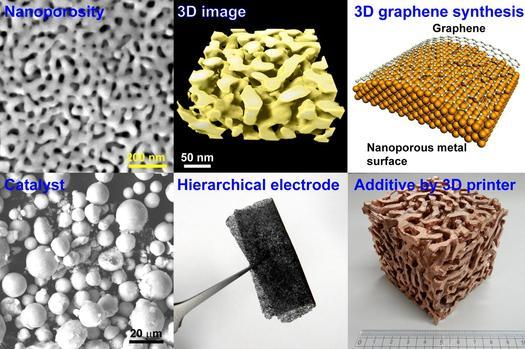

## 1. Introduction

Dealloying refers to selective leaching of one or more components out of a solid solution alloy or compound to produce a residual nanoporous structure [1]. Recently, dealloying has been utilized as a facile method for fabricating nanoporous metals containing the three-dimensional (3D) bicontinuous structure characterized by open nanopores with tunable sizes. Hence, dealloyed nanoporous metals represent a new class of functional materials with large surface areas and unique structural properties such as mechanical rigidity, high electrical conductivity, and high corrosion resistance.

Interestingly, dealloying gold-copper alloy surfaces with natural acids (which is also called depletion gilding) was used in the ancient Incan Empire for coloring gems to shine gold [2]. As another famous example, Raney nickel was obtained by dealloying nickel-aluminum alloy in 1927 [3], but its microstructure and the related pore formation mechanism were not sufficiently studied at that time. First, Pickering and Swann investigated gold alloy corrosion via transmission electron microscopy (TEM) and obtained a nanoporous structure with pore sizes of around 10 nm [4,5]. Second, A.J. Forty [6] reported a TEM image of nanoporous gold (NPG) fabricated by dealloying AuAg alloy with nitric acid in *Nature* magazine in 1979 and proposed a possible mechanism of nanopore formation [6], but his study has not attracted much attention for a long time. The dealloying technique was noticed by the scientific community when Erlebacher et al. [7] proposed an atomistic model for nanoporosity evolution in *Nature* after conducting molecular dynamics simulations and studying the kinetics and potential dependence of the dealloying process, owing to the growing popularity of nanotechnology and nanoscience during that period (in 2001). As a result, subsequent studies in that area led to the creation of cross-interdisciplinary research fields related to batteries, catalysis, sensing, and biotechnology applications because nanoporous metals with excellent chemical and physical properties could be fabricated by this facile method [8–10].

**Figure 1.**
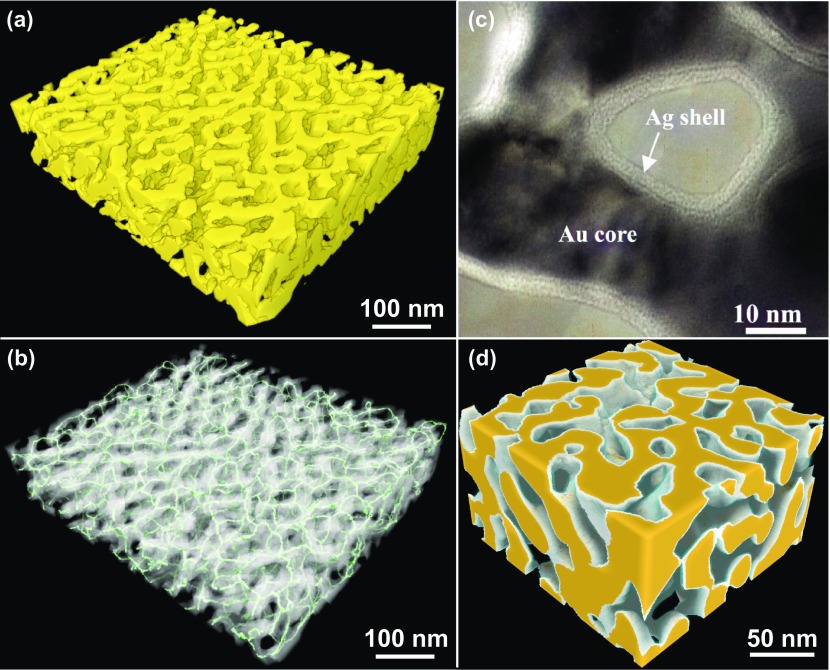
(a) A 3D image of NPG. (b) Skeletal network of the gold ligaments generated by using a 3D thinning algorithm. (c) A magnified TEM micrograph of the silver-plated NPG. (Adapted with permission from Ref. [32]. Copyright (2008) American Chemical Society.) (d) A schematic diagram of the nanoporous metal core-shell structure. (Adapted with permission from Ref. [63]. Copyright (2009) American Chemical Society.).

Using various dealloying techniques, many novel nanoporous metals have been produced. Thus, nanoporous Ag [11], Pt [12,13], Pd [14], Ir [15], Ni [16–18], Cu [19,20], Ti [21], Ru [22], and Au alloy [23] were fabricated by dealloying AgAl, PtAl, PdCo, IrMg, NiMn, CuMn, TiSc, and RuMn alloys and multicomponent metallic glasses, respectively. Among these materials, NPG represents the most widely studied prototype of dealloyed nanoporous metals and one of the nanomaterials which can be easily fabricated in the chemical laboratory of a junior high school. Because inexpensive white gold leaves (12 carat, 50 wt.% Ag) are commonly available in leaf shops, their simple etching with nitric acid can be conducted at minimal cost [24]. The pore sizes of the resulting materials were systematically studied by varying the dealloying conditions, such as acid concentration, applied potential [25], dealloying temperature [26], annealing treatment [27], and reaction environment [28–30]. The dealloying process is controlled by the diffusion of gold atoms at the alloy/electrolyte interface. Naturally low dealloying temperatures can significantly reduce the interfacial diffusivity of gold atoms and produce ultrafine NPG with a pore size of 5 nm [26] (its representative 3D image visualized by electron tomography is displayed in Figure 1(a)). The skeletonized inter-connected gold ligaments, which exhibit a bicontinuous network, are shown in Figure 1(b). This metallic bicontinuous network distinguishes NPG from many other fragmented nanomaterials by its enhanced electrical conductivity and mechanical stability, which make it highly suitable for electrode fabrication. Interestingly, the analysis of 3D objects revealed that their area distributions of the convex and concave surfaces were identical; hence, their total mean curvature was equal to zero (this phenomenon is also observed for minimal surfaces in mathematics such as gyroids) [31]. Owing to their bicontinuous structure, various core-shell nanoporous composites were fabricated using simple electrochemical and surface functionalization techniques such as electroplating, electroless plating, atomic layer deposition, and chemical vapor deposition (CVD). They included Au(core)/Ag(shell) [32,33], Cu/Au [34], Au/Pt [35–39], Au/TiO_2_ [40–42], Au/Al_2_O_3_ [43], Au/MnO_2_ [44,45], Au/SnO_2_(Sn) [46], Au/RuO_2_ [47], Au/MoS_2_ [48,49], Au/polypyrrole [50–52], and Au/polyaniline [53–55] systems. As an example, a TEM image of the Au/Ag core-shell nanoporous composite with an Ag shell thickness of 2 nm is shown in Figure 1(c) [32], while its 3D schematic is depicted in Figure 1(d). Thus, not only monolithic nanoporous metals, but also nanoporous composites embedded in other materials (metals, oxides, sulfides, and polymers) can be easily produced for various applications.

In this short review, we focus on the recent achievements describing the intriguing functionalities of nanoporous metals/composites as green materials. In particular, such areas as molecular detection, catalysis, 3D graphene synthesis, and hierarchical pore formation are discussed in detail, and the possibility of using these materials for additive manufacturing (AM) performed via 3D printing is evaluated. Finally, we propose feasible ways of realizing the ultimate 3D functionality of organic-inorganic hybrids beyond the scope of nanoporous metals.

## 2. Versatile applications

### 2.1. Molecular detection

Surface-enhanced Raman scattering (SERS) is a surface-sensitive technique where Raman scattering is enhanced by the molecular adsorption on rough metallic nanostructures via electromagnetic and chemical enhancement mechanisms [56]. The chemical mechanism usually leads to the SERS enhancement with a factor of 10^2^–10^3^ depending on the orbital interactions between the metals and the adsorbed molecules, where charge transfer likely occurs during low-energy resonant Raman scattering [57]. The electromagnetic mechanism characterized by a Raman enhancement factor of more than 10^10^ results from the excitation of surface plasmons in metallic nanostructures, which generates intense local electric fields (‘hot spots’) that strongly affect the optical properties of the adsorbate. Therefore, a major part of the SERS enhancement occurs via the electromagnetic mechanism. In order to detect individual molecules using a SERS-based technique, silver colloidal nanoparticles randomly deposited on glass or silicon substrates are usually utilized to tailor the size and population of the ‘hot spots’ [58,59]. However, the main disadvantage of using silver nanoparticles is the poor reproducibility of their shapes and population on the substrate surface as well as the easy degradation in air due to oxidation.

**Figure 2.**
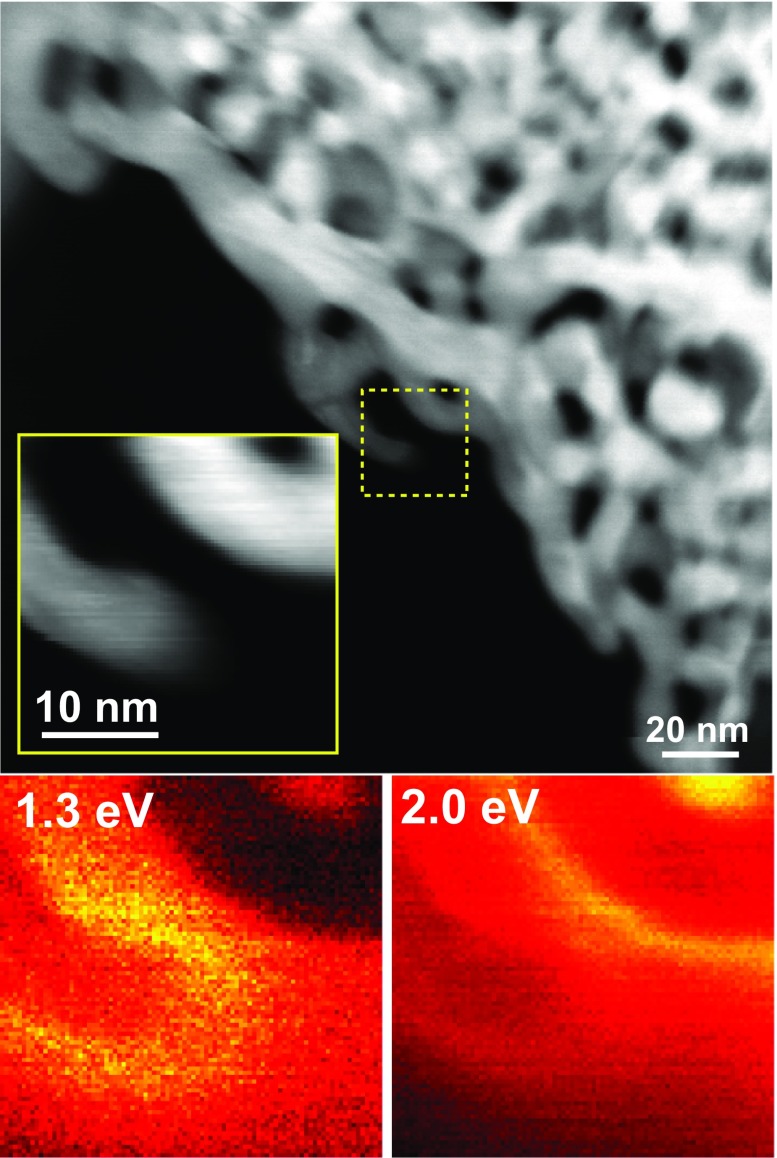
A STEM image of NPG obtained via low energy-loss electron spectroscopy. The region of interest (displayed in the inset) is denoted by the yellow square. The energy-loss images obtained at 1.3 and 2.0 eV are shown in order to visualize the corresponding surface plasmon energies.

**Figure 3.**
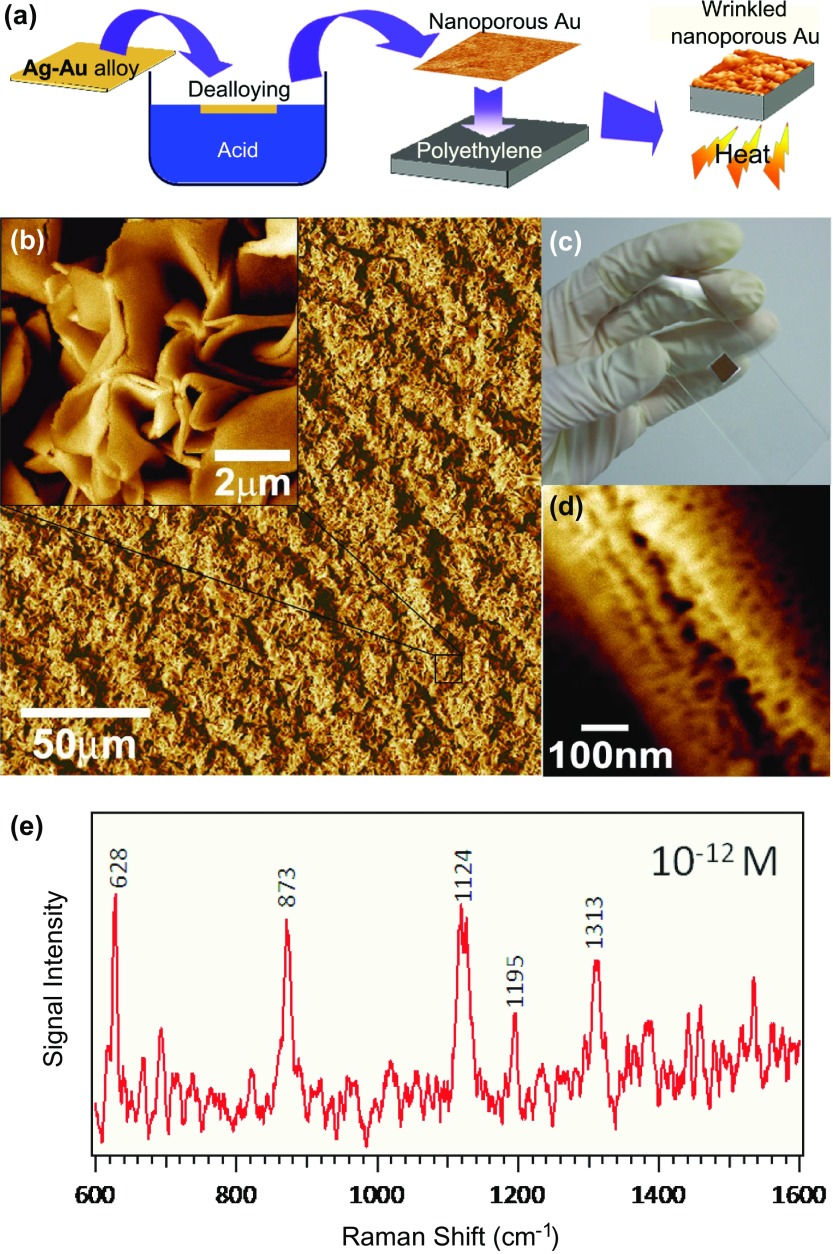
(a) A schematic describing the preparation of wrinkled nanoporous films. (b) A microstructure of the wrinkled nanoporous film with a quasi-periodic wavelength of 10–15 μm. (c) A photograph of the wrinkled nanoporous film with dimensions of 8 mm × 8 mm. (d) A microstructure of the wrinkled ridges containing nanogaps, interleaving broken ligaments, and linear chains of self-similar nanocavities. (e) A single-molecule SERS spectrum of 10^-12^ M adenine solution with selected Raman bands. (Adapted from Ref. [62]. Copyright Liu et al. (2011)).

After taking into account the difficulty of controlling the nanoparticle properties, freestanding NPG films can be considered facile substrates for SERS applications due to their tunable nanoporosity as well as the excellent chemical stability and biocompatibility of gold. For example, the SERS effect produced by the rhodamine 6G molecules adsorbed on the NPG surface with pore sizes ranging from 5 nm to 700 nm (which were tailored by the combination of low-temperature dealloying and annealing) has been systematically investigated [27]. The obtained results reveal that the strongest SERS enhancement was achieved for the sample with an ultra-fine nanopore size of around 5 nm since the pores with smaller sizes produced larger signals. To elucidate the mechanism of the SERS enhancement, Lang et al. [60] investigated the observed electromagnetic distribution via discrete dipole approximation simulations and concluded that the enhancement phenomenon mainly resulted from the presence of localized surface plasmons in nanoscaled Au ligaments and electromagnetic coupling between the neighboring ligaments (these plasmons can be detected using the energy-loss electron spectroscopy module of the TEM instrument due to the relatively small energy resolution of its electron beam (<0.3 eV); see Figure 2). In general, the energy (frequency) distribution of surface plasmons depends strongly on the nanostructure geometry. For the NPG sample depicted in Figure 2, a plasmon with an energy of 1.3 eV is localized near the tip of the Au ligament, while another plasmon with an energy of 2.0 eV is localized near the neighboring ligament. Because of the presence of the randomly nanostructured Au network, such ‘hot spots’ with various plasmon frequencies (corresponding to a wide range of molecular detection) are uniformly distributed across the entire NPG film. To further enhance the observed SERS effect of ‘hot spots’, wrinkled NPG films were obtained by the thermal contraction of the underlying pre-strained polystyrene substrates (see Figure 3(a)) [61]. Figure 3(b) shows scanning electron microscopy (SEM) images of the wrinkled NPG films on the glass substrates obtained at low and high magnifications, respectively. Figure 3(c) shows the photograph of the wrinkled nanoporous film with dimensions of 8 mm × 8 mm. As shown in Figure 3(d), these films contain a large number of cracks with nanogaps and nanotips along their edges, which significantly enhance the local electric fields. By further optimizing the Ag contents in NPG films, a detection limit of 10^−12^ M DNA adenine was ultimately achieved, which corresponded to the single-molecule detection level (see Figure 3(e)) [62].

Since gold is one of the most expensive precious metals, the use of a nanoporous non-noble metal decorated with a thin layer of a noble metal should be considered for optical and catalytic applications. Owing to the potential applications of copper as an inexpensive raw material, nanoporous copper (NPC) was fabricated by dealloying Cu_30_Mn_70_ alloy [19]. The systematic investigation of the obtained material revealed that the SERS inertness of Mn suppressed the SERS enhancement at very small nanopore sizes due to the increased residual concentration of Mn. In order to further improve the SERS performance of NPC, silver shells with tunable thicknesses were deposited *in situ* on the Cu ligament surfaces via a facile hydrometallurgical route [63]. The resultant nanoporous composites exhibited significant improvement in their SERS properties by a factor of 16 as compared to those of the as-prepared NPC. Thus, biocompatible nanoporous metals with superior reproducibility and excellent chemical stability, which can be easily fabricated via a facile synthesis procedure, represent ideal SERS substrates for a wide range of applications in the fields of life science and environmental protection where molecule detection and identification are of critical importance.

### 2.2. Catalysis

Gold is considered a chemically inert material because its standard electrode potential is the highest among the metals (it is still possible to hear in the news that the ancient gold coins manufactured many centuries ago and recently found inside a sunken ship have retained their tantalizing gold color). However, gold nanoparticles deposited on oxide supports were found to be very active for CO oxidation at temperatures as low as -70 °C by Haruta et al. [64] in 1987. The reason for the observed catalytic behavior of Au nanoparticles has been debated since that time, and the results of recent studies strongly suggest that the nanoparticle edges as well as the interface between the gold and the oxide phases contain active sites [65–67]. According to the proposed mechanism, the CO molecules adsorbed on gold particles migrate toward the perimeter of the support oxide surface and react with adsorbed oxygen to form bidentate carbonate species. The detailed history of the studies on Au nanoparticles and role of the perimeter interfaces in their catalytic activity are summarized elsewhere [68,69].

**Figure 4.**
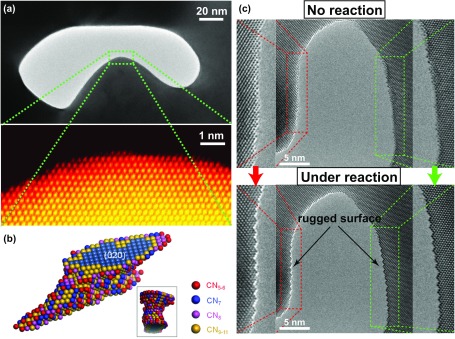
(a) TEM (upper panel) and STEM (lower panel) images of NPG obtained at low and high magnifications, respectively. (b) Reconstructed 3D atomic configuration of the NPG ligament. Different colors denote the surface atoms with different coordination numbers. (c) An environmental TEM image of the rugged surface obtained during CO oxidation and in the absence of any reactions. Panels (a) and (c) are adapted from Ref. [72]. © Macmillan Publishers Limited, Springer Nature. Panel (b) is adapted from Ref. [73]. © WILEY-VCH Verlag GmbH & Co. KGaA, Weinheim.

NPG contains no oxides or perimeter interfaces; however, it was also found to be catalytically active towards CO oxidation [70,71], and the origin of its catalytic properties has been debated as well. In this respect, the NPG microstructure was characterized via spherical aberration-corrected (Cs-corrected) TEM [72]. The atomic structure of the internal NPG surfaces (which are responsible for its catalytic activity) was examined using a scanning transmission electron microscope (STEM) equipped with a high-angle annual dark-field (HAADF) detector. The arrangement of the near-surface atoms was also determined with high accuracy by Cs-corrected high-resolution TEM (HRTEM), which provided phase-contrast images with high displacement sensitivity. From the results of extensive HRTEM and STEM characterizations, it was suggested that the superior catalytic performance of NPG originated from the surface strain and high concentration of low-coordination atoms stabilized by the complex geometry of bicontinuous NPG nanopores (see Figure 4(a)). After conducting STEM imaging along the [001], [101], and [112] zone axes of the face-centered cubic lattice of NPG ligaments, its reconstructed 3D atomic configuration was visualized by discrete tomography [73]. The surface atoms with different coordination numbers were marked by various colors in Figure 4(b) to highlight the existing surface defects. As indicated by the results of statistical analysis, the fraction of the under-coordinated surface atoms on NPG ligaments was much larger than that on the truncated octahedral gold particles with identical diameters. These under-coordinated NPG surface atoms represent geometrically necessary surface defects and are kinetically more stable than the nanoparticle surface atoms. In addition to the static observations in an inert vacuum environment, we also characterized the evolution of the NPG atomic surface structure during the CO oxidation in a reactive atmosphere using a newly developed environmental HRTEM technique (Figure 4(c)). The NPG sample containing 1.2 at% of Ag begins to exhibit catalytic activity along with significant surface reconstruction at a pressure of several Pa (in addition, the {111} faceting dynamics along with the {011} surface can be clearly observed from the {011} direction [72]). The obtained results indicate that the low-coordination atoms on the topmost NPG surface with a relatively high density are involved in the CO oxidation reaction as the catalytically active sites and represent the main source for the formation of the {111} facets. Since the residual Ag atoms may also play an important role in the CO oxidation reaction, a NPG sample containing around 20 at% of the residual Ag species with similar pore sizes has been fabricated. Interestingly, the {111} faceting dynamics is significantly suppressed during CO oxidation as compared to the process observed for the low-Ag NPG in the same environment, indicating that the presence of residual Ag atoms significantly stabilizes the surface steps and kinks of NPG. In summary, both the under-coordinated Au atoms and residual elements such as Ag appear to play important roles by providing high-energy binding sites for CO adsorption and catalytically active sites for the activation of molecular oxygen, respectively [74].

Similar to the homogeneous Au catalysts, NPG was also utilized in heterogeneous organic reactions conducted under liquid-phase conditions. In 2010, Asao et al. [75] reported for the first time the use of NPG for the conversion of silanes to silanols in acetone at ambient temperatures (other important organic reactions that can utilize NPG as a catalyst include semihydrogenation, hydrosilylation, diboration of alkynes, and benzannulation [76]). More recently, NPG has been used as a highly chemoselective hydrogeneration agent for the reduction of terminal alkynes in the presence of internal alkynes, which could not be achieved previously using supported gold nanoparticle catalysts [77]. The utilization of nanoporous copper also led to a significant enhancement of catalytic processes in the click chemistry without using any supports or bases [78]. In addition, nanoporous Pd, which was fabricated by dealloying Pd_30_Ni_50_P_20_ metallic glass, exhibited remarkable catalytic activity for the Suzuki-coupling reaction between iodoarenes and arylboronic acids under mild conditions as well as for the Heck reaction of versatile aryl iodides and aryl bromides [79,80].

**Figure 5.**
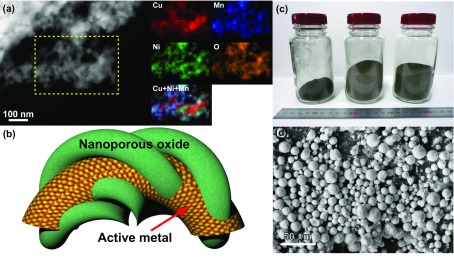
(a) Microstructures of nanoporous CuNiMnO after NO reduction durability testing. The obtained STEM image and EDS chemical maps of the selected area show the distributions of Cu (red), Ni (green), Mn (blue), and O (orange) elements. (b) A schematic diagram of the new concept of catalyst design, in which the active metal is tangled with the nanoporous oxide. The described system exhibits higher density of the perimeter interfaces as compared to that in the conventional nanoparticle/oxide system. (c) A photograph of the mass-produced nanoporous CuNiMnO catalysts. (d) An SEM image of the gas-atomized micropowders after dealloying. Panels (a), (c), and (d) adapted from Ref. [82]. © WILEY-VCH Verlag GmbH & Co. KGaA, Weinheim.

Gas conversion in automobiles relies on the use of precious metal catalysts and rare-earth oxide supports fabricated from Pt, Pd, and CeO_2_. Lately, the consumption of Pt has grown significantly to meet the increasing demands of ground transportation in developing countries and various technologies utilized in developed countries such as fuel cells and water splitting [81]. Hence, earth-abundant nanoporous CuNiMnO catalyst has been produced in our group by leaching Mn from CuNiMn alloy precursor [82]. The resulting material was durable and catalytically active toward both NO reduction and CO oxidation. During catalytic reactions, its nanostructure self-transformed into a more active nanostructure with very active Cu/CuO regions, and its further significant coarsening was not accompanied since these regions were tangled with a stable nanoporous NiMnO network (see Figure 5(a)). This self-transformed nanostructure successfully completed a long-term durability test for 10-d NO reduction at 400 °C, and the TEM observations conducted *in situ* also provided evidence for its instantaneous reaction-induced transformation. A new concept of catalyst design drawn from this study is illustrated in Figure 5(b). The active metal layer tangled with nanoporous oxide is characterized by a high density of its perimeter interfaces as compared to those of the conventional nanoparticle/oxide systems. The monotonic nanopores do not exhibit sufficient heat resistance to suppress the pore degradation via coarsening; however, in this new design, despite the coarsening of the active metal regions to sizes exceeding 100 nm, they can preserve their perimeter interfaces as long as they remain inside the stable nanoporous oxide network. In the past, the mass production of nanoporous catalysts was rarely feasible. By now, cold-rolled foils, leaves, and melt-spun ribbons have been used as precursor alloys for basic studies; however, we found that the micro-powders with particle sizes below 50 μm produced by a gas-atomizing method are suitable for mass production. We demonstrated the large-scale fabrication of nanoporous CuNiMnO catalyst by leaching Cu_15_Ni_15_Mn_70_ gas-atomized powders (see Figures. 5(c) and (d)). The use of microalloyed powders can decrease the dealloying time and achieve more efficient productivity, which also provides additional ideas of material design toward realizing the ultimate 3D functionality (which will be discussed below).

The reaction-driven transformation of porous nanostructures into active nanostructures is not limited to metals, but can also be applied to intermetallic and alloy compounds. Recently, Ni_3_Nb has evolved into a nanophase-separated structure consisting of the filamentous Ni networks with thicknesses below 10 nm incorporated into a niobium oxide matrix, whose nanostructure is capable of sustaining the thermal agglomeration during long-term NO reduction conducted at elevated temperatures [83].

### 2.3. 3D graphene synthesis

**Figure 6.**
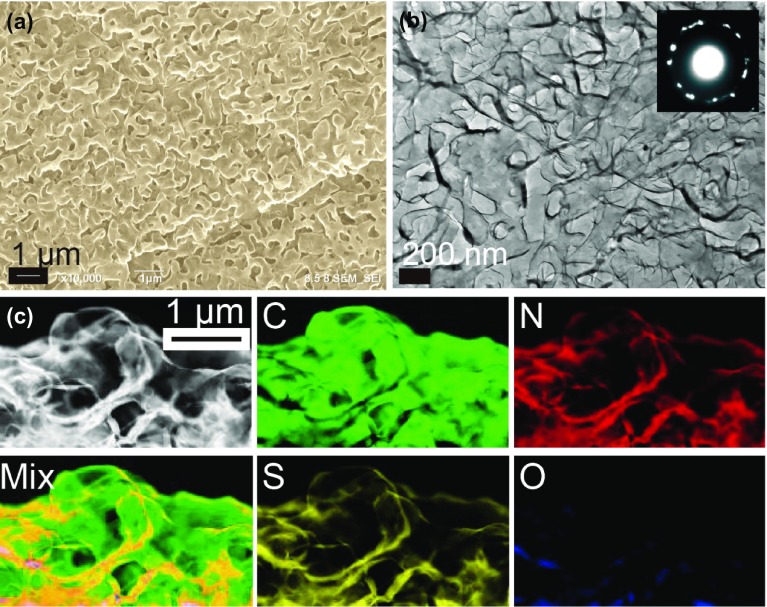
(a) and (b) Representative SEM and TEM images of the 3D nanoporous graphene, respectively. (c) EELS chemical maps of the C, N, and S elemental distributions in the doped nanoporous graphene. (Adapted from Ref. [90]. © Wiley-VCH Verlag GmbH & Co. KGaA, Weinheim.).

Graphene represents a one atom thick sheet of graphite containing sp^2^-hybridized carbon atoms arranged in a hexagonal honeycomb lattice, which was isolated in 2004 by Andre Geim and Konstantin Novoselov using what was colloquially called a ‘scotch tape’ technique [84,85]. Graphene intrinsically exhibits higher electron mobility as compared to that of any other known materials (which amounts to 200,000 cm^2^⋅V^−1^⋅s^−1^) due to the presence of massless particles called Dirac fermions [86]. CVD is an inexpensive and facile method for the deposition of high-quality graphene layers on transition metal substrates such as Ni, Pd, Ru, Ir, and Cu [87,88]. However, converting two-dimensional (2D) graphene sheets into stacked 3D graphene is not easy without decreasing its high electron mobility. Hence, high-quality 3D graphene was successfully synthesized by combining the CVD technique and templates fabricated from nanoporous transitional metals (such as Ni) [89]. The nanoporous Ni has a bicontinuous structure consisting of a smooth robust surface and nanopores. Thus, uniform graphene films were grown across the nickel templates after heating in the CVD furnace filled with hydrogen, argon, and benzene gases. Subsequently, the nanoporous nickel template was removed by etching in acid, yielding a freestanding bicontinuous 3D structure of nanoporous graphene (its representative SEM and TEM images are shown in Figures 6(a) and (b), respectively [90]). Interestingly, the 3D nanoporous graphene can preserve the distinctive 2D coherent electronic properties, as indicated by the results obtained via photoemission spectroscopy. After scaling its magnetotransport properties, which was performed using a semiclassical theory, the estimated carrier mobility of 3D graphene networks was equal to about 5000–7500 cm^2^ V^−1^ s^−1^, which was comparable to those of the CVD-grown flat 2D graphene sheets [91]. More recently, strong tunable plasmonic absorption in the terahertz to mid-infrared regime with controllable doping levels and pore sizes was realized for fabricating novel plasmonic sensors due to the presence of intrinsic 2D Dirac plasmons in the 3D nanoporous graphene [92].

Graphene by itself exhibits little catalytic activity because of its high chemical stability. Using the developed CVD technique for graphene growth, chemically active nanoporous graphene can be fabricated by replacing the benzene precursor with other compounds such as pyridine (C_5_H_5_ N), thiophene (C_4_H_4_S), and triphenylphosphine (Ph_3_P) for N, S, and P doping, respectively [90,93,94]. It can potentially be used in electrocatalysis as a cathode electrode for the oxygen-reduction reactions of fuel cells [94]. The other potential application of nanoporous graphene is replacing the Pt electrode utilized in the hydrogen evolution reaction (HER) in acid solutions for water splitting. Accordingly, the N and S co-doped nanoporous graphene was successfully used for the HER reaction, and the uniform distribution of dopants at the sub-nanoscale was confirmed via STEM-electron energy loss spectroscopy (EELS) analysis, as shown in Figure 6(c) [90]. As indicated by the results of *ab initio* calculations, the dopant atoms were stabilized at the geometrically necessary structural defects of the hexagonal graphene lattice to preserve its porous structure. The obtained surface area was 800 m^2^/g, which was 500 times larger than that of flat graphene. Therefore, in the HER experiments, the onset potential of -0.2 V was able to maintain a much larger current density (as compared to that of flat graphene) due to the abundance of pores for mass transport and reactions occurring on both their internal and external surfaces that maximized the utilization of the available pore space. Currently, by further optimizing the amount of starting material for the nanoporous Ni temperate, contents of N, S, and P dopants as high as 6.4% were achieved [93]. Such large amounts of synergistic dopants in the 3D nanoporous graphene structure resulted in a high density of active sites and, therefore, its superior catalytic performance toward high-efficiency electrochemical hydrogen production. Moreover, the nanoporous graphene containing single-atom nickel dopants exhibited superior HER catalytic properties in a 0.5 M H_2_SO_4_ solution with a low overpotential of approximately 50 mV and Tafel slope of 45 mV dec^−1^ as well as excellent cycling stability [95]. Owing to these advantages, freestanding nanoporous graphene sheets can be directly installed into rechargeable Li-O_2_ batteries as feasible and economic cathode materials [96]. More recently, high-performance Li-O_2_ batteries characterized by stable cycling at large capacities and low charge potentials have been fabricated in the forms of coin cells and pouches using tetrathiafulvalene redox additive and dimethyl sulfoxide-based electrolyte [97]. In particular, the gravimetric capacity and energy density of the produced large-sized Li-O_2_ pouch batteries exceed those of the commercial Li-ion batteries.

**Figure 7.**
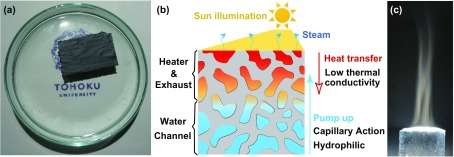
Steam generation by a thin porous graphene sheet. (a) An optical image of the piece of porous N-doped graphene sheet. (b) A schematic description of the heat localization system, which converts sunlight into steam using a piece of porous N-doped graphene as the steam generator. Thin porous graphene plays a versatile role in harvesting solar illumination as thermal energy. (c) An optical image of the steam generation process, in which the scrap from panel (b) is placed under the concentrated solar illumination. (Adapted from Ref. [98]. © Wiley-VCH Verlag GmbH & Co. KGaA, Weinheim).

The 3D nanoporous graphene does not exhibit the same light permeability as 2D graphene does, and its color is naturally black as indicated by the optical micrograph depicted in Figure 7(a) [98]. The black object under solar illumination can be considered an economic heat source for generating high-energy steam since water heating via solar illumination is becoming the most common global application of solar energy. The materials utilized for heat localization must possess low specific heat, effective light absorption, low thermal conductivity, and open mesoscopic capillary porosity. Ideally, a single material should exhibit all the required thermal, optical, and wetting characteristics to avoid the interference from composite interfaces. By varying the level of nitrogen doping, pore size, and pore thicknesses, a single piece of porous N-doped 3D graphene with a thickness of around 35 μm can satisfy all the requirements and realize the conversion of sunlight to high-energy steam via heat localization at a high energy efficiency of 80% (in contrast to the magnitude of 53% obtained for the conventional graphite powders) because its sub-micrometer-sized pore channels are suitable for the capillary action of water. Moreover, nitrogen doping decreases the thermal conductivity of porous graphene, which is related to the bandgap widening via chemical doping and enhanced graphene wettability (the mechanism of steam generation by heat localization is described in Figures 7(b) and (c)). Such multifunctional abilities of the N-doped 3D graphene can be applied to the new utilization of sunlight and sewage purification for environmental cleanup [98]. Thus, the open porous structure of 3D graphene characterized by bicontinuous porosity, tunable pore sizes, large surface area, high electron mobility, and tunable dopant levels can be widely used for various physical and chemical applications.

### 2.4. Hierarchical pore formation

**Figure 8.**
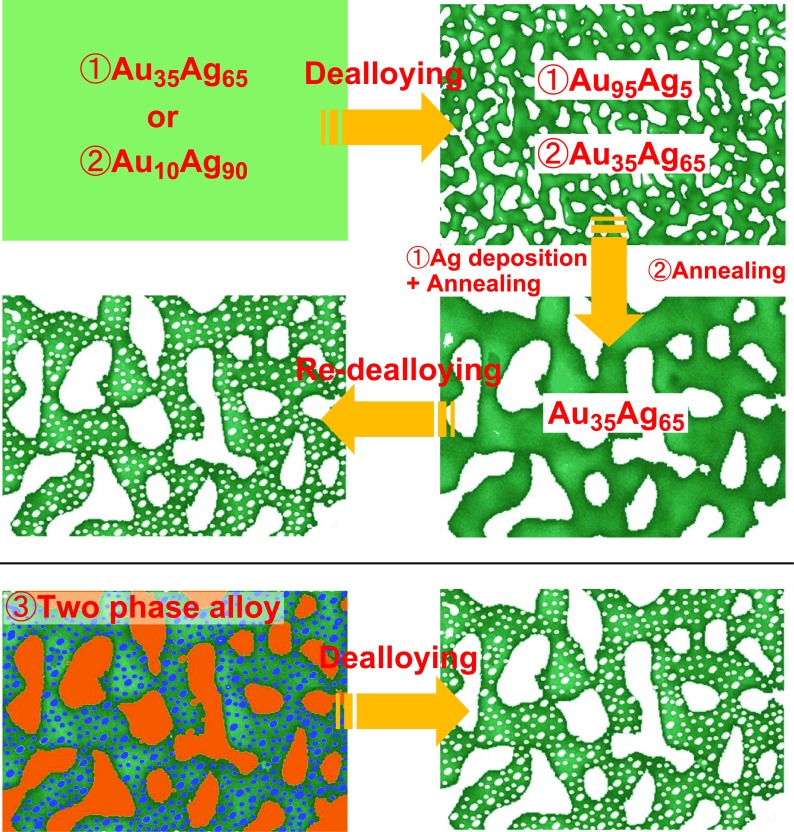
Three basic strategies (1–3) of the hierarchical pore fabrication via dealloying. The details of the corresponding procedures are explained in the main text.

**Figure 9.**
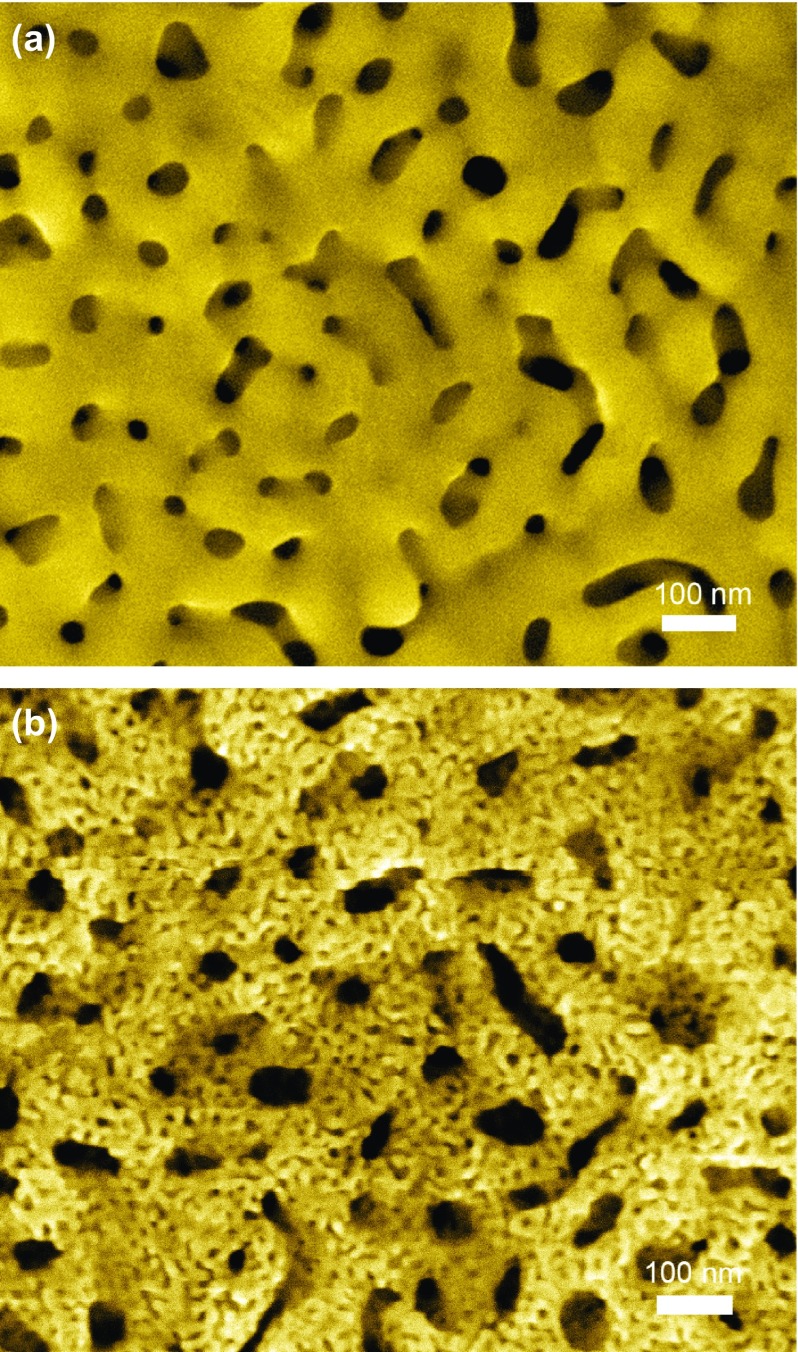
SEM images of the hierarchical NPG. (a) Coarsened nanoporous AuAg alloy with a pore size of 80–100 nm. (b) Hierarchical NPG particles containing small pores with diameters of 5–20 nm on the surface of ligaments with sizes of 80–100 nm. (Adapted from Ref. [102]. Copyright Guo et al (2016)).

Dealloyed nanoporous metals usually have unimodal pore sizes. Since nanopores (characterized by diameters below 1 μm) impede mass transport, and micropores (with sizes greater than 1 μm) can promote fast mass exchange, a hierarchical structure with various pore sizes can be utilized for smooth mass transport combined with a large surface area. Previously, some dealloying strategies have been proposed for building a hierarchical (bi-modal) porous structure (three of them are illustrated in Figure 8). Ding and Erlebacher [99] demonstrated the following dealloying/plating/re-dealloying technique. First, regular etching of an Au_35_Ag_65_ leaf was performed under corrosion-free conditions (corresponding to its floating on the surface of a concentrated nitric acid solution for 1 hour) followed by heat treatment. As a result, a structure with unimodal pore sizes of approximately 200–300 nm and residual Ag content of less than 5% was obtained. Next, an Ag layer was electrochemically deposited on the NPG surface, and NPG foil with relatively large pores was prepared by the second annealing (the latter re-homogenized the gold/silver elements, simultaneously increased the pore sizes, and produced the Ag-rich NPG structure). The final dealloying step dissolved the Ag species in the re-homogenized gold/silver alloy ligaments. Subsequently, to avoid the complicated Ag deposition procedure described by Ding and Erlebacher [99], a new alloy precursor with a higher Ag content (Ag_90_Au_10_) was prepared (similar Ag-rich precursor alloys corresponding to 6 and 9 carat gold are commercially available in Italy and Germany). The initial short-term corrosion of such material creates a nanoporous Ag-rich Au alloy (similar to Au_35_Ag_65_), which is then thermally coarsened to form a porous alloy with pore sizes of around 200 nm corresponding to the upper hierarchical level. The second dealloying step removes the Ag species completely to reach a lower hierarchical level in the form of nanopores inside the larger ligaments [100,101]. The representative SEM images obtained before and after the final dealloying steps of the second strategy are displayed in Figures 9(a) and (b), respectively [102]. Finally, the bi-modal nanoporous structure can also be fabricated by dealloying two-phase precursors. Zhang et al. [103] produced an Al-Au precursor alloy containing the primary Al phase and Al_2_Au intermetallic phase via rapid solidification. Al_2_Au dendrites first precipitated during the rapid solidification process followed by the formation of the α-Al solid solution phase from the remaining liquid. During dealloying, the fast excavation of the α-Al phase contributed to the formation of large-sized channels, and the slow dealloying of the Al_2_Au phase produced a nanoporous structure.

**Figure 10.**
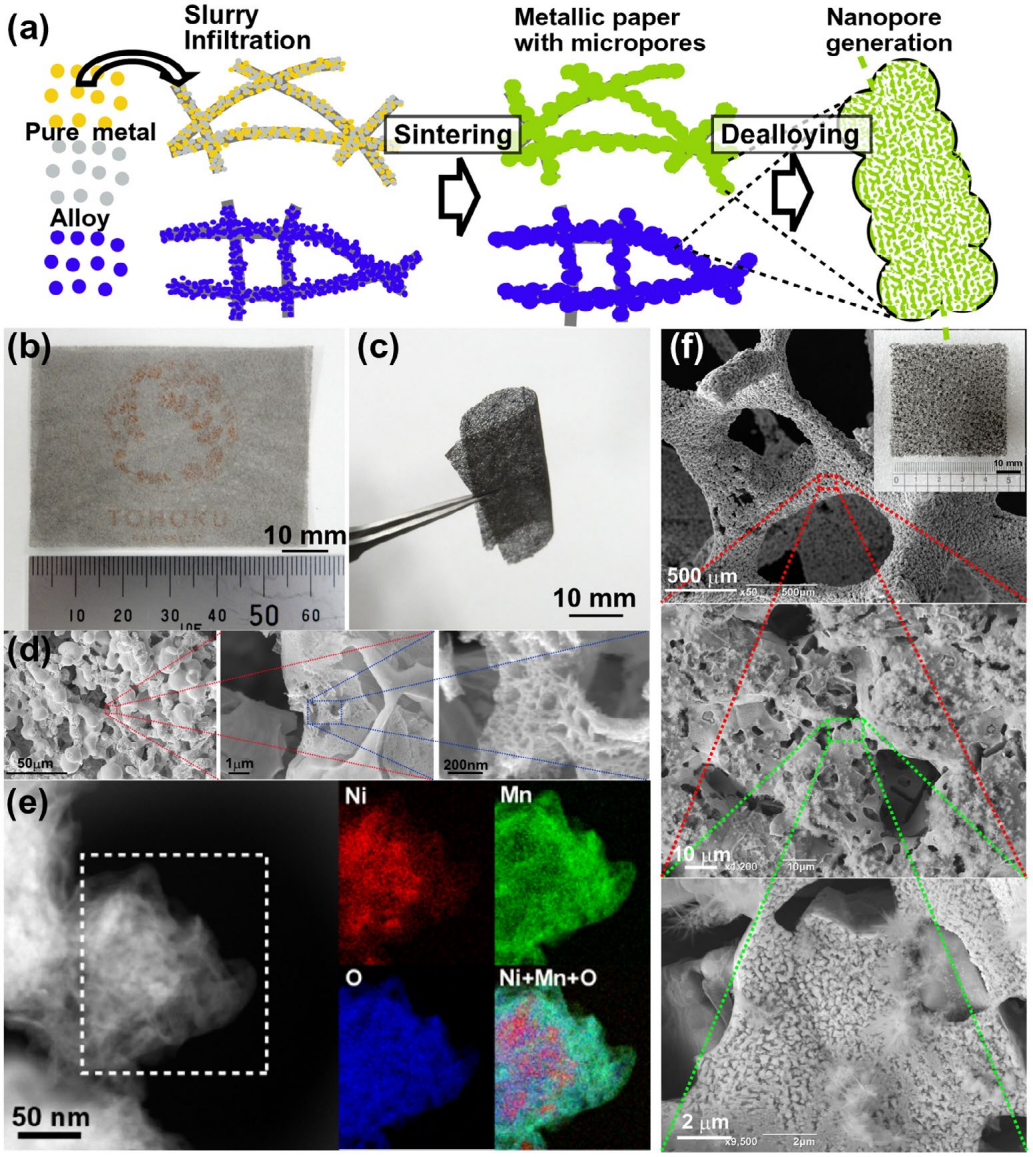
(a) A schematic describing the creation of a hierarchical porous structure by sintering a slurry of metal powder mixed with water-soluble binder on a paper sheet. (b) A 50 μm thick Ni_30_Mn_70_ alloy sheet (the logo of Tohoku University is located on the reverse side). (c) A bendable thin dealloyed Ni_30_Mn_70_ alloy sheet. (d) SEM images of the Ni_30_Mn_70_ microporous nanostructure obtained after dealloying. (e) STEM image and chemical maps of the selected area containing the distributions of Ni (red), Mn (green), and O (blue) elements. (f) SEM images of the Cu_15_Ni_10_Fe_5_Mn_70_ microporous metal alloy (shown in the inset) obtained after dealloying. (Panels (a)–(e) are adapted from Ref. [104]. Copyright Fujita et al. (2015)).

Both the first and second strategies require the use of multiple electrochemical processes that are too costly and time-consuming for commercial-scale production. In the third strategy, a bi-modal nanoporous structure can be fabricated from two-phase precursors via one-step dealloying, but this approach also limits the utilized metal systems to two-phase precursors. Therefore, we have developed an innovative combination of the advanced powder metallurgy and dealloying to create hierarchical porous Au-Ag and Ni-Mn electrodes using Japanese ‘Washi’ paper as a template [104]. As a result, a bi-modal porous structure was produced from metallized papers via one-step dealloying, while a tri-modal porous structure was fabricated for the first time through the two-step dealloying of Au-Ag. According to this strategy (illustrated in Figure 10(a)), pure metal and alloy powders are first prepared and collected by water atomization and filtering. After that, a template material consisting of cellulosic fibers with diameters less than 50 μm (Japanese ‘Washi’ paper) is filled with a slurry of metal powder (characterized by particle sizes of less than 5 μm) and water-soluble binder (the complete covering of the template surface is ensured by the capillary action). During high-temperature sintering, both the template material and carbon-based binder are decomposed, while the remaining metal powder becomes consolidated to form a sheet. If different pure metal powders are used in the slurry (such as Au and Ag ones), a solid solution alloy can be created. The described sintering technique is applicable to most metal powders and can be used for the net-shape mass production of ultra-thin microporous metal sheets. The subsequent one-step dealloying procedure removes the less noble elements, indicating that nanoscale pores can be created in metal sheets with pre-existing microscale porosity. The thin NiMn alloy sheet depicted in Figure 10(b) was sufficiently transparent to see the logo of Tohoku University printed on its reverse side with a high degree of mechanical flexibility retained even after the dealloying step (see Figure 10(c)). SEM images of the hierarchical nanoporous NiMn structure with a residual Mn content of around 20 at% are shown in Figure 10(d) (finer nanopores can be observed at a higher magnification). The results of TEM imaging revealed the presence of well-developed oxide compounds such as nickel hydrate Ni(OH)_2_ and manganese oxide (see Figure 10(e)). Such oxides are typically not observed in the images of conventional nanoporous Ni; hence, this phenomenon can be explained by the increase in the size of the micropores located between ligaments, which provides additional space for structural development. The BET surface area of the produced structure increased to 101 m^2^/g, which represents the highest value ever reported for nanoporous Ni. Because of its developed nanostructure, the hierarchical nanoporous NiMn electrode exhibits high electrochemical capacitance and oxygen evolution reaction activity. Instead of using ‘Washi’ paper, other commercially available templates (such as urethane) can be utilized for fabricating thick and robust microporous metals (see the SEM image depicted in Figure 10(f)). The Cu_15_Ni_10_Fe_5_Mn_70_ microporous metal displayed in the inset was industrially manufactured using a urethane template. After one-step dealloying, its hierarchical pore structure was realized. We believe that the described combination of the microporous alloy fabrication via powder metallurgy with subsequent dealloying can be easily applied to other alloy systems for the development of sensing devices, catalysts, and energy storage/conversion systems followed by their manufacture on an industrial scale.

### 2.5. Additive manufacturing via 3D printing

The AM technique (known as 3D printing) has been developing very rapidly. It allows building 3D objects from powder, wires, or sheets in a layer-by-layer manner. Today, the related methods for manufacturing metal objects are represented by selective laser melting or electron beam melting. Sames et al. [105] compare various metal AM techniques, and their strengths and limitations are discussed. The main drawback of the AM approach is a very limited number of applicable alloy systems; thus, suitable commercially available materials include steels, stainless steels, structural aerospace material (Ti–6Al–4V), bio-compatible implant materials (such as Ti, Ti–6Al–4V, and CoCr), and high-temperature materials (Inconel 626, Inconel 718) for high-impact industries. Another drawback of metal AM is its very high input costs (including those of the hardware, feedstock, and maintenance), which currently limit its scope of applications to academic researchers. Owing to recent advancements in this area, a novel AM system that is capable of processing different metal powders simultaneously to produce ‘colorful’ objects has been developed (but not yet commercialized) by JEOL [106], while the majority of other AM systems can only use one type of metal powders to fabricate ‘monotone’ objects.

**Figure 11.**
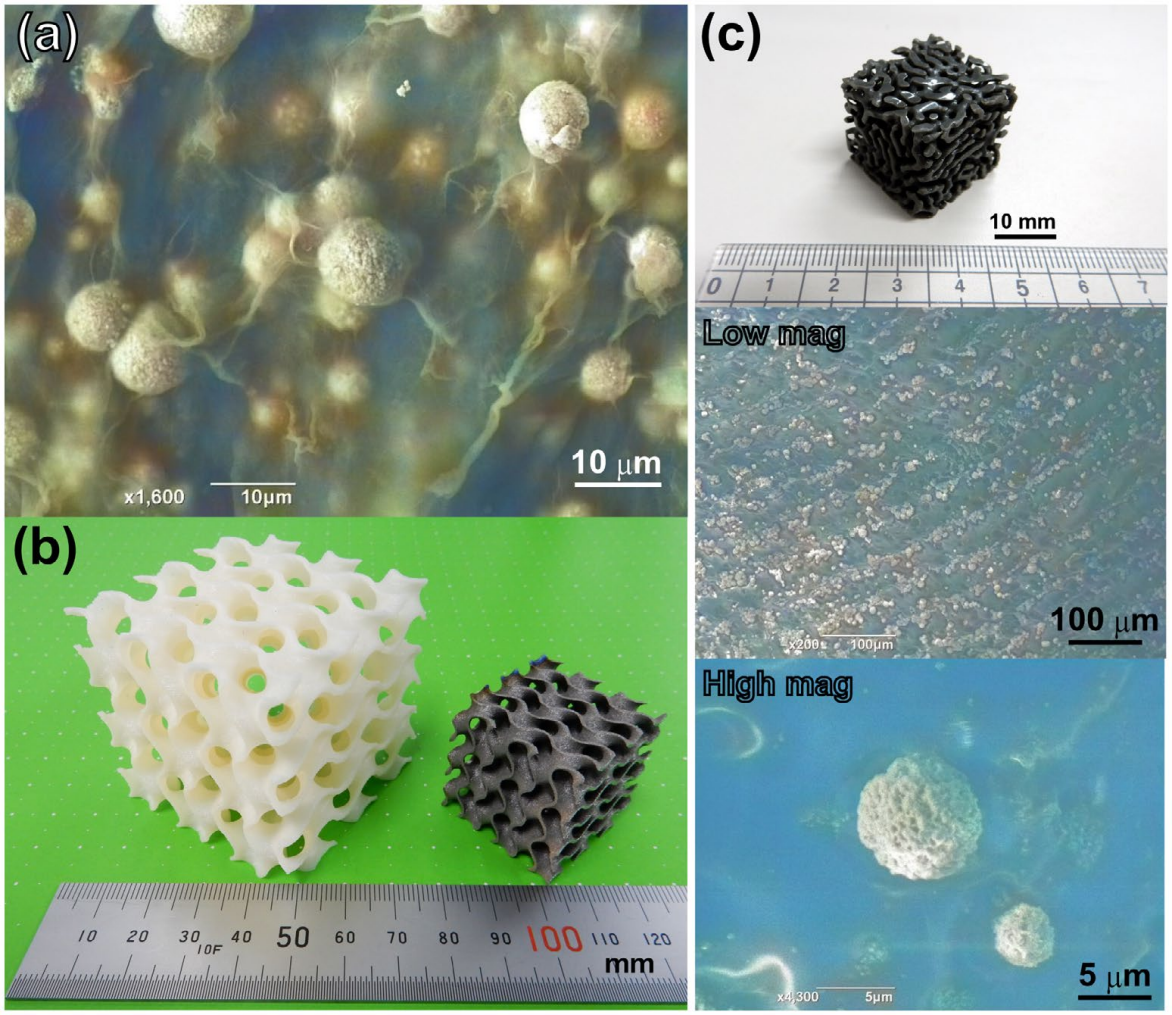
(a) A SEM image of the nanoporous Cu microspheres dispersed among the cellulose nanofibers. (b) A photograph of the gyroid shapes fabricated from acrylonitrile butadiene styrene (ABS) resin (left) and Ti–6Al–4V alloy (right). (c) A photograph of the randomly selected porous ABS resin composited with dispersed nanoporous Cu microspheres. The SEM surface images were obtained both at low and high magnifications.

As has been discussed above, the dealloyed gas-atomized nanoporous microsphere can be considered a functionalized microstructural ‘element’ for many purposes; therefore, it can also be added to virtually any object. For example, Cu_30_Mn_70_ spherical powder was mixed with a commercially available dispersion of cellulose nanofibers; as a result, a piece of paper containing embedded nanoporous copper spheres was fabricated after the subsequent drying and etching steps, as demonstrated in Figure 11(a). Such paper can be further transformed into a novel 3D shape using either an origami or kirigami technique, which has been extensively studied in recent years [107,108]. In order to evaluate the ability of the modern AM techniques to fabricate porous objects, a gyroid-type porous structure was obtained from Ti–6Al–4V alloy, as shown in Figure 11(b) (the gyroid shape was selected since it possessed a unique pore structure and could be described by the simple mathematical equation sin*x*con*y* + sin*y*cos*z* + sin*z*cos*x* = 0). Unfortunately, we were unable to utilize our alloy powders instead of Ti alloys for the external metal AM systems because they could cause serious contamination (it is virtually impossible to use the newly developed alloy powders without owning an expensive AM system with the initial cost of more than $1 million). However, despite the extremely high costs of the modern metal AM systems (which significantly exceed the typical budget of an academic researcher), other AM-related techniques such as stereolithography (SL) can be used. The SL method involves the layer-by-layer conversion of liquid resins into solid cross-sections using an ultraviolet laser and continues to remain one of the most widely used rapid prototyping techniques for plastic models. Therefore, we have attempted to mix our prepared Cu_30_Mn_70_ alloy powders with a liquid resin to fabricate a structure mimicking random porous features via SL; as a result, a porous copper microsphere was clearly observed on the surface of the etched specimen, as shown in Figure 11(c). We are currently investigating possible inexpensive AM methods for fabricating solid hierarchical porous structures for various applications.

## 3. Perspectives

Following a long trail from ancient times to modern civilization, nanoporous metals have been established as a novel class of materials. In this review, we have discussed the attractive features of nanoporous metals as well as the achievements of our research group in this field. However, many unexplored topics remain to be investigated. For example, dealloying by metallic melt was reported by Wada et al. [109] in 2011. This technique can be applied to the preparation of nanoporous less noble metals and non-metals such as Ti [109], Nb [110], Si [111], Fe [112], and Cr [112] as well as graphite [113], ferritic stainless steel (Fe-Cr) [112], and β-Ti (Ti-Cr-Zr) alloy [114], whose dealloying mechanism is described in detail elsewhere [115]. In particular, the nanoporous Si obtained by this method can be used for fabricating high-quality anodes with a high lithium capacity approaching its theoretical limit and significantly extended service lives, which are comparable with those of the electrodes manufactured from silicon nanoparticles [111,116]. Moreover, NPG is an extraordinarily well-defined model system for studying the mechanical behavior of metal nanostructures, and its deformation mechanism as well as the ligament size effect on the yield strength have been extensively investigated both experimentally and theoretically [117].

**Figure 12.**
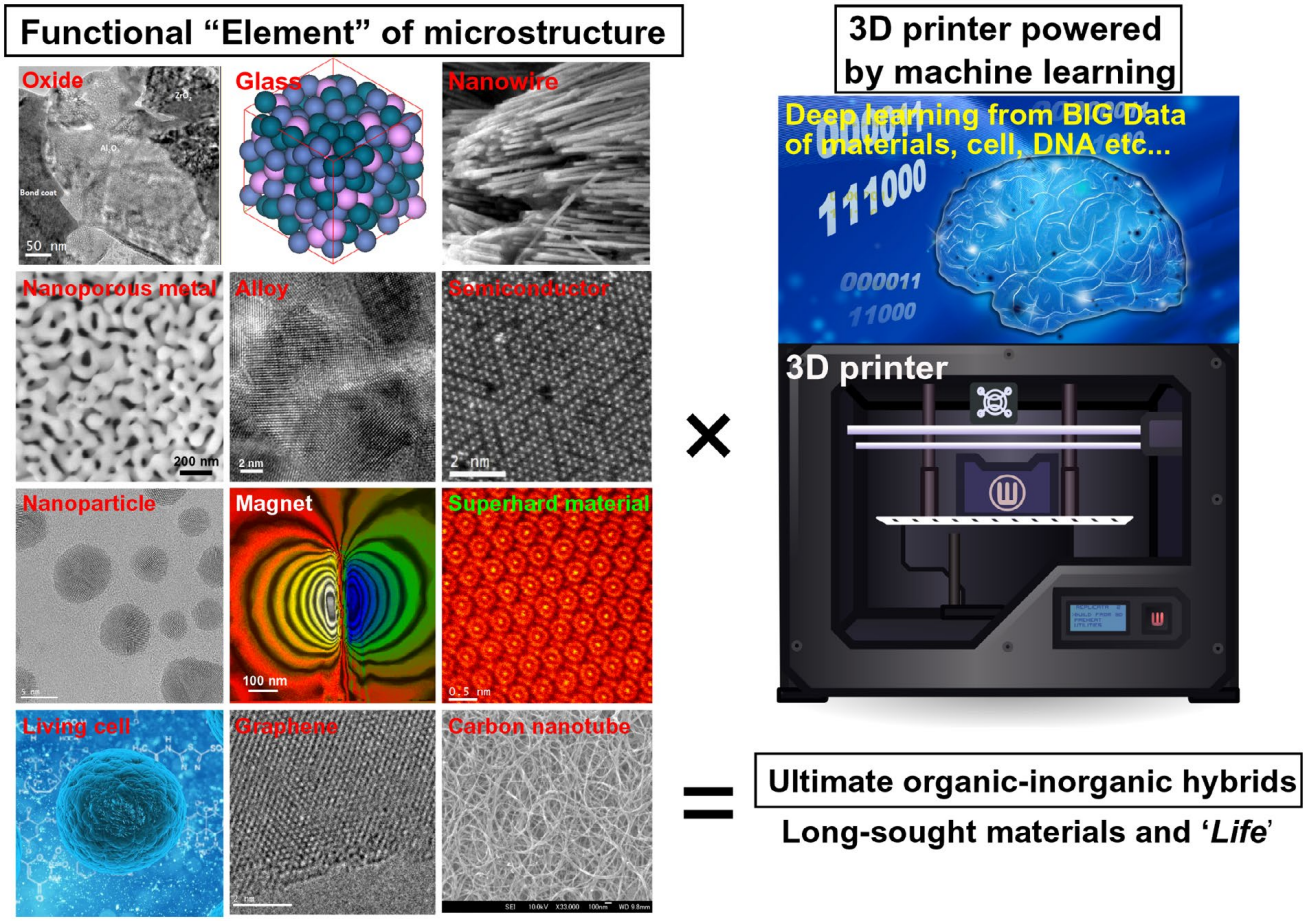
Future perspective: toward achieving the ultimate 3D functionality based on the concept of the ‘functional elements of microstructure’, which is similar to periodic tables. A super 3D printing technology powered by the ultimate AI might discover the long-sought organic-inorganic hybrids that represent ‘life’.

The future outlook toward achieving the ultimate 3D functionality is summarized in Figure 12. Similar to periodic tables, it is very interesting to note that the nanoporous microsphere dealloyed from gas-atomized powders can be considered a mere example of functional ‘elements’ among many other novel microstructures (including living cells). Various characteristic microstructures (including those of superalloys, superconductors, shape memory alloys, and metallic glasses) are widely known, and their powders with unique functionalities for 3D printers can be fabricated. The development of the 3D printing technology for medicine is proceeding very rapidly, and multiple powders/cells will be able to be simultaneously integrated with high precision very soon. The key issues here are the selection of a proper method for designing 3D objects with desired properties and the exact positioning of the required functional elements in the resulting 3D structure. A possible solution to these problems can be obtained using recent advances in the field of information technology. Similar to 3D printing technology, the fields of superior artificial intelligence (AI) [118,119], neuromorphic devices [120,121], and data informatics (represented by the ‘big data’ collected for various materials, cells, and DNA [122,123]) are also progressing beyond our current level of knowledge. It remains an open question: what and how can the ultimate AI learn from the Big Data design via AI-driven 3D printing? Similar to our human body containing hierarchical blood vessels, the AI may design a combination of hierarchical nanoporous metals for fluid channels, catalysts, electrolytes, cooperative cells, and self-heating ceramics to ultimately create an artificial life (AL) in the future: in other words, ‘AI begets AL’.

## Funding

This study was mainly supported by the JST-PRESTO program ‘New Materials Science and Element Strategy’ and research funds provided by the ‘World Premier International (WPI) Research Center Initiative for Atoms, Molecules and Materials’, MEXT (Japan). It was also partially supported by KAKENHI [grant numbers JP15K13796, JP16H02293, and JP17H06220] and the JST-CREST program ‘Innovative Catalysts and Creation Technologies for the Utilization of Diverse Natural Carbon Resources’ [grant number JPMJCR15P1].

## Disclosure statement

No potential conflict of interest was reported by the author.
